# Gastrointestinal cancer occurs as extramuscular manifestation in FSHD1 patients

**DOI:** 10.1038/s10038-022-01095-0

**Published:** 2022-11-07

**Authors:** Takashi Kurashige, Hiroyuki Morino, Hiroki Ueno, Tomomi Murao, Tomoaki Watanabe, Takao Hinoi, Ichizo Nishino, Tsuyoshi Torii, Hirofumi Maruyama

**Affiliations:** 1 Department of Neurology, National Hospital Organization Kure Medical Center and Chugoku Cancer Center, Kure, Japan; 2 Department of Clinical Neuroscience and Therapeutics, Hiroshima University Graduate School of Biomedical and Health Sciences, Hiroshima, Japan; 3 Department of Medical Genetics, Tokushima University Graduate School of Biomedical Sciences, Tokushima, Japan; 4 Division of Clinical and Molecular Genetics, Hiroshima University Hospital, Hiroshima, Japan; 5 Department of Neuromuscular Research, National Institute of Neuroscience, National Center for Neurology and Psychiatry, Kodaira, Japan

**Keywords:** Epidemiology, Neuromuscular disease

## Abstract

Facioscapulohumeral dystrophy type1 (FSHD1) patients with a shortened D4Z4 repeat containing the *DUX4* gene have a broad spectrum of clinical manifestations. In addition, high expression of DUX4 protein with an aberrant C terminus is frequently identified in B cell acute lymphoblastic leukemia. We investigated clinical manifestations in 31 FSHD1 patients and 30 non-affected individuals. Gastrointestinal cancers (gastric and colorectal cancers) increased after the age of 40 years and were more frequently observed in FSHD1 patients (*n* = 10) than in non-affected individuals (*n* = 2, *p* = 0.0217), though the incidence of cancers occurring in non-gastrointestinal tissues of FSHD1 patients was the same as that of non-affected individuals (*p* > 0.999). These comorbidities of FSHD1 patients were not associated with D4Z4 repeat number. Our results suggest that gastrointestinal cancers are among the extramuscular manifestations of adult FSHD1 patients, and do not depend on D4Z4 repeat number.

Facioscapulohumeral dystrophy (FSHD) is one of the most frequent muscular dystrophies and typically affects the facial, scapulohumeral, tibial, and axial muscles [1, 2]. The most frequent cause of FSHD is partial loss of D4Z4 microsatellite repeats in the subtelomere of chromosome 4q (FSHD type 1 [FSHD1]; OMIM 158900). FSHD1 patients have only 1 to 10 of these D4Z4 repeats, whereas healthy individuals have 11 or more [3]. Loss of D4Z4 repeats results in partial D4Z4 chromatin relaxation as microsatellite repeat instability (MSI) and derepression of muscular DUX4 [4]. Muscular DUX4 in turn activates a series of transcriptional programs that eventually leads to muscle cell death [4].

The FSHD1 phenotype encompasses a broad spectrum of severity ranging from nonpenetrant mutation carriers to severely affected patients. It is estimated that up to 21% of patients show symptoms in childhood [5], and those symptoms are varied [6–11]. In comparison, the clinical spectrum of adult FSHD patients is not fully known except for deafness. DUX4 is known to work as an oncogenic driver causing B cell acute lymphoblastic leukemia in adolescents and young adults [12], which suggests that DUX4 also works as an oncogenic driver in the organs of FSHD1 patients.

In this study, we aimed to characterize extramuscular symptoms of adult FSHD1 patients clinically and genetically. We interviewed 31 FSHD1 patients of Hiroshima University Hospital and the National Hospital Organization Kure Medical Center and Chugoku Cancer Center and retrospectively collected their clinical data, including age at onset, D4Z4 repeat number, present functional mobility, age at loss of ambulation, and comorbidities including diabetes mellitus, hyperlipidemia, cardiovascular events, and cancers. The shortened repeat and haplotype of D4Z4 repeat were confirmed by Southern blotting. We also interviewed 30 non-affected (non-FSHD1) family members of FSHD1 patients and collected their medical history including present functional mobility and comorbidities. All values are expressed as mean ± standard deviation (SD) unless stated otherwise. Differences among means were analyzed with the Kruskal–Wallis test, Mann–Whitney test, or chi-square test. These analyses were performed in the Prism 7 software (GraphPad Software, La Jolla, CA). This study was approved by the institutional ethical committees of Hiroshima University Hospital and National Hospital Organization Kure Medical Center and Chugoku Cancer Center and performed after obtaining written informed consent from the patients and families.

The clinical characteristics of FSHD1 patients and non-affected individuals are summarized in Table 1. With regard to age, FSHD1 patients (47.5 ± 16.7 years old) and non-affected individuals (51.1 ± 18.8 years old) did not show a significant difference (*p* = 0.7228). Meanwhile, the proportion of females was higher in non-affected individuals (M:F = 9:21) than in FSHD1 patients (M:F = 19:12, *p* = 0.0209). The age at onset of FSHD1 was 14.5 ± 3.5 years old and the disease duration was 35.1 ± 13.3 years. Among FSHD1 patients, 12 could walk without support, 9 used supportive devices to walk, and 10 could not walk. Five male FSHD1 patients and 7 male non-affected individuals had smoking history (*p* = 0.0166) though females of two groups did not have. We could not fully collect drinking histories of FSHD1 patients and non-affected individuals.

**Table 1:** The clinical characteristics of FSHD1 patients and non-affected individuals

	FSHD1 patients [Age at onset, mean ± SD]	non-affected individuals [Age at onset, mean ± SD]	*P* value
Number of participants (M:F)	31	30	
Age (y.o., mean ± SD)	48.9 ± 15.1	51.1 ± 18.8	0.7228
Gender (M:F)	19:12	9:21	0.0209
Age at onset (y.o., mean ± SD)	14.5 ± 3.5	n.a.	n.a.
Disease duration (y.o., mean ± SD)	35.1 ± 13.3	n.a.	n.a.
Walk without support	12/31 (38.7%)	30/30 (100%)	<0.0001
Walk with supportive devices	9/31 (29.0%) [44.0 ± 8.9]	0	
Lost of ambulation	10/31 (32.3%) [54.6 ± 6.9]	0	
Smoking	5/31 (16.1%)	7/30 (23.3%)	0.5339
Male smoker	5/19 (26.3%)	7/30 (77.8%)	0.0166
Deafness	11/31 (35.5%) [30.5 ± 10.9]	0	n.a.
Retinopathy	3/31 (9.7%) [35.3 ± 15.7]	0	n.a.
Diabetes mellitus	10/31 (32.3%) [46.8 ± 11.9]	0	n.a.
Hyperlipidemia	18/31 (58.1%) [44.0 ± 9.5]	0	n.a.
Cardiovascular events	8/31 (25.8%) [56.4 ± 17.8]	0	n.a.
Cardiomyopathy	6/31 (19.4%) [60.3 ± 22.1]	0	n.a.
All Cancers	10/31 (32.3%) [52.6 ± 17.1]	2/30 (6.7%) [44.5 ± 12.0]	0.0217
Gastrointestinal cancers (Gastric and colorectal cancers)	10/31 (32.3%) [52.6 ± 17.1]	2/30 (6.7%) [49.5 ± 4.9]	0.0217
Colorectal cancers	8/31 (25.8%) [56.4 ± 18.0]	2/30 (6.7%) [49.5 ± 4.9]	0.0807
Colorectal cancer among smokers	4/5 (80.0 %) [52.3 ± 23.4]	1/7 (14.3%) [53.0]	
Other cancers	1/31 (3.2%) [70.0] (Endometrial cancer)	1/30 (3.3%) [36.0] (Endometrial cancer)	>0.9999

We also analyzed the clinical data of FSHD1 patients according to their D4Z4 repeat number (Table 2). Three patients had 2 repeats of D4Z4, 15 patients had 3 repeats, and 13 patients had 4 repeats. The age at onset was highest in patients with 2 repeats (*p* = 0.0466), because one such patient only became aware of his symptoms at 24 years old. There were no significant differences in disease duration with respect to repeat number. The proportion of ambulatory patients was significantly associated with repeat number (*p* = 0.0091).

**Table 2:** The clinical characteristics and *D4Z4* repeat number of FSHD1 patients

	2 repeats	3 repeats	4 repeats	*P* value
Number of patients (M:F)	3 (2:1)	15 (10:5)	13 (7:6)	0.8587
Age (y.o., mean ± SD)	58.0 ± 13.9	50.1 ± 16.4	46.9 ± 13.6	0.4738
Age at onset	19.3 ± 4.2	14.5 ± 3.4	13.3 ± 2.5	0.0466
Disease duration	38.7 ± 11.2	35.6 ± 15.2	33.6 ± 12.1	0.6898
Walk without support	0 (0.0%)	3/15 (20.0%)	8/13 (61.5%)	0.0091
Walk with supportive devices	3/3 (100.0%)	4/15 (26.7%)	3/13 (23.1%)	
Lost of ambulation	0 (0.0%)	8/15 (53.3%)	2/13 (15.4%)	
Smoking	2/3 (66.7%)	2/15 (13.3%)	1/13 (7.7%)	0.0401
Male smoker	2/2 (100%)	2/10 (20.0%)	1/7 (14.3%)	0.0423
Age at using devices (y.o., mean ± SD)	48.3 ± 5.5	40.7 ± 8.8	49.2 ± 8.1	0.1102
Age at using wheelchairs (y.o., mean ± SD)		55.5 ± 7.4	51.0 ± 4.2	0.5333
Deafness	2/3 (66.7%)	8/15 (53.3%)	1/13 (7.7%)	0.0152
AAO of deafness (y.o., mean ± SD)	29.0 ± 9.9	30.8 ± 12.2	31.0	0.9819
Retinopathy	0 (0.0%)	3/15 (20.0%)	0 (0.0%)	0.1532
AAO of retinopathy (y.o., mean ± SD)		35.3 ± 8.1		n.a.
Diabetes mellitus	0 (0.0%)	8/15 (53.3%)	2/13 (15.4%)	0.0361
AAO of diabetes mellitus (y.o., mean ± SD)		46.3 ± 4.7	49.0 ± 7.1	0.6222
Hyperlipidemia	2/3 (66.7%)	11/15 (73.3%)	5/13 (38.1%)	0.1159
AAO of hyperlipidemia (y.o., mean ± SD)	48.5 ± 4.9	42.1 ± 8.0	46.4 ± 4.6	0.5059
Cardiovascular events	0 (0.0%)	6/15 (40.0%)	2/13 (15.4%)	0.1606
AAO of cardiovascular events (y.o., mean ± SD)		56.8 ± 6.8	55.0 ± 2.8	>0.9999
Cardiomyopathy	0 (0.0%)	5/15 (33.3%)	1/13 (7.7%)	0.1076
AAO of cardiomyopathy (y.o., mean ± SD)		61.0 ± 6.9	57.0	n.a.
Cancer	2/3 (66.7%)	5/15 (33.3%)	3/13 (23.1%)	0.2996
AAO of cancers (y.o., mean ± SD)	57.0 ± 4.2	49.2 ± 17.4	55.3 ± 3.2	0.8770
Gastrointestinal cancer	2/3 (66.7%)	5/15 (33.3%)	3 (23.1%)	0.2933
AAO of gastrointestinal cancer (y.o., mean ± SD)	57.0 ± 4.2	49.2 ± 17.4	55.3 ± 3.2	0.8770
Colorectal cancer	2/3 (66.7%)	4/15 (26.7%)	2 (15.4%)	0.1606
AAO of colorectal cancer (y.o., mean ± SD)	57.0 ± 4.2	56.0 ± 9.8	56.5 ± 3.5	0.9857
Endometrial cancer	0 (0.0%)	1/15 (6.7%)	0 (0.0%)	n.a.
AAO of endometrial cancer (y.o., mean ± SD)		70.0		n.a.

*AAO* age at onset

Regarding extramuscular symptoms, 11 FSHD1 patients were aware of deafness, and three had retinopathy. Non-FSHD1 individuals did not have any medical history of diabetes mellitus, hyperlipidemia, or cardiovascular events. Meanwhile, among FSHD1 patients, 10 patients had diabetes mellitus, which was observed within their fifth and sixth decades (Fig. 1A). Hyperlipidemia was observed in 18 FSHD1 patients, and the number with hyperlipidemia increased after the age of 30 years (Fig. 1B). Eight patients had a medical history of cardiovascular events; of those, 6 were treated for their cardiomyopathies and 2 suffered from ischemic strokes. These cardiovascular events also appeared within the patients’ fifth and sixth decades (Fig. 1C). Of the various comorbidities, deafness was associated with D4Z4 repeat number (*p* = 0.0152). Diabetes mellitus, hyperlipidemia, and cardiovascular events were not associated with D4Z4 repeat number.

**Fig. 1:**
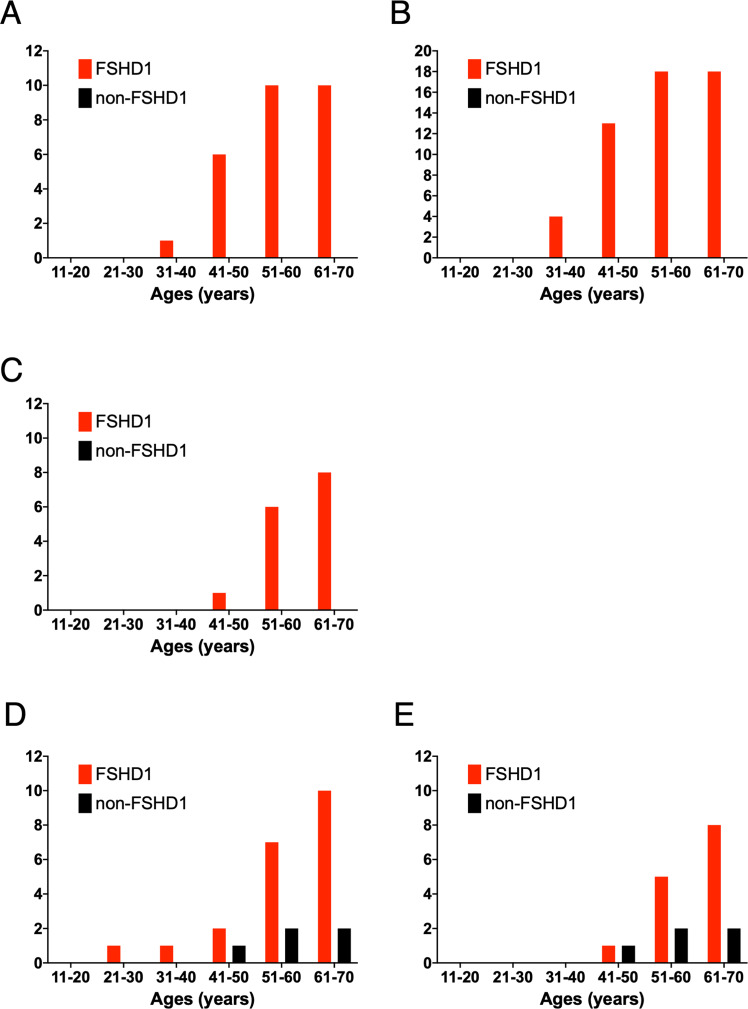
Box plot and cumulative frequency histogram of the onset age of extramuscular symptoms in FSHD1 patients and non-affected (non-FSHD1) individuals. **A** Among FSHD1 patients, 10 patients had diabetes mellitus within their fifth and sixth decades, while none did among non-FSHD1 individuals. **B** Hyperlipidemia was observed in 18 FSHD1 patients, but not in non-FSHD1 individuals. The number of FSHD1 patients with hyperlipidemia increased after the age of 30 years. **C** Cardiovascular events occurred in 8 FSHD1 patients in their fifth and sixth decades, but not in non-FSHD1 individuals. **D** Gastrointestinal cancers (gastric and colorectal cancers) also increased after the age of 40 years and were more frequently observed in FSHD1 patients (10 patients vs 2 non-FSHD1 individuals). **E** Colorectal cancers were observed in eight FSHD1 patients and two non-affected individuals after the age of 40 years. However, there was no significant difference in the number of colorectal cancers

The number of patients with cancer increased after the age of 40 years, and was higher in FSHD1 patients (*n* = 10, 32.3 %) than in non-affected individuals (*n* = 2, 6.7 %, *p* = 0.0217). Interestingly, all patients with cancer had gastrointestinal cancers (gastric and colorectal cancers) (Fig. 1D). Among patients at least 40 years of age, colorectal cancers were observed in eight FSHD1 patients and two non-affected individuals (Fig. 1E). One FSHD1 patient and one non-affected individual suffered from both colorectal and endometrial cancers. Meanwhile, there was no difference in the incidence of cancers occurring in non-gastrointestinal tissues between FSHD1 patients (*n* = 1) and non-affected individuals (*n* = 1, *p* > 0.999). Among 10 FSHD1 patients with cancer, only one FSHD1 patient died due to colorectal cancer, which was as low as the incidence of cancer death of FSHD patients previously reported [13].

FSHD1 patients harbor a partial loss of D4Z4 microsatellite repeats, which results in muscular cell death via partial D4Z4 chromatin relaxation and muscular DUX4 activation [4]. The incidence of gastrointestinal cancer was not associated with D4Z4 repeat number, but rather with the presence of any partial loss of the D4Z4 repeat. Our FSHD1 patients with cancers did not satisfy the Bethesda criteria for HNPCC because their onset ages were over 50 years.

In conclusion, we identified gastrointestinal cancers, cardiovascular events, diabetes mellitus, and hyperlipidemia as extramuscular manifestations in adult FSHD1 patients. FSHD1 patients had a higher risk of gastrointestinal cancer, which did not depend on D4Z4 repeat number. Gastrointestinal screening on a regular basis is therefore necessary for elderly FSHD1 patients to prevent gastrointestinal cancers.
